# Responses of leaf morphology, NSCs contents and C:N:P stoichiometry of *Cunninghamia lanceolata* and *Schima superba* to shading

**DOI:** 10.1186/s12870-020-02556-4

**Published:** 2020-07-29

**Authors:** Qingqing Liu, Zhijun Huang, Zhengning Wang, Yanfang Chen, Zhumei Wen, Bo Liu, Mulualem Tigabu

**Affiliations:** 1 Forestry College, Fujian Agriculture and Forestry University, Fuzhou, 350002 Fujian People’s Republic of China; 2 Fujian Provincial Colleges and University Engineering Research Center of Plantation Sustainable Management, Fuzhou, 350002 Fujian People’s Republic of China; 3 Southern Swedish Forest Research Center, Faculty of Forest Science, Swedish University of Agricultural Sciences, PO Box 49, SE-230 53 Alnarp, Sweden

**Keywords:** *Cunninghamia lanceolate*, Light adaptation, Non-structural carbohydrate, Soluble sugar, Starch

## Abstract

**Background:**

The non-structural carbohydrates (NSCs), carbon (C), nitrogen (N), and phosphorus (P) are important energy source or nutrients for all plant growth and metabolism. To persist in shaded understory, saplings have to maintain the dynamic balance of carbon and nutrients, such as leaf NSCs, C, N and P. To improve understanding of the nutrient utilization strategies between shade-tolerant and shade-intolerant species, we therefore compared the leaf NSCs, C, N, P in response to shade between seedlings of shade-tolerant *Schima superba* and shade-intolerant *Cunninghamia lanceolate*. Shading treatments were created with five levels (0, 40, 60, 85, 95% shading degree) to determine the effect of shade on leaf NSCs contents and C:N:P stoichiometry characteristics.

**Results:**

Mean leaf area was significantly larger under 60% shading degree for *C. lanceolata* while maximum mean leaf area was observed under 85% shading degree for *S. superba* seedlings, whereas leaf mass per area decreased consistently with increasing shading degree in both species. In general, both species showed decreasing NSC, soluble sugar and starch contents with increasing shading degree. However shade-tolerant *S. superba* seedlings exhibited higher NSC, soluble sugar and starch content than shade-intolerant *C. lanceolate*. The soluble sugar/starch ratio of *C. lanceolate* decreased with increasing shading degree, whereas that of *S. superb* remained stable. Leaf C:N ratio decreased while N:P ratio increased with increasing shading degree; leaf C:P ratio was highest in 60% shading degree for *C. lanceolata* and in 40% shading degree for *S. superba*.

**Conclusion:**

*S. superba* is better adapted to low light condition than *C. lanceolata* through enlarged leaf area and increased carbohydrate reserves that allow the plant to cope with low light stress. From mixed plantation viewpoint, it would be advisable to plant *S. superba* later once the canopy of *C. lanceolata* is well developed but allowing enough sunlight.

## Introduction

Non-structural carbohydrates (NSCs, mainly composed of soluble sugars and starch) are important energy source for all plant growth and metabolism [1, 2]. Soluble sugars are photosynthesis products and are used to meet plant current requirements and osmotic regulation [3]. Starch is the main form of energy stores and is used to meet plants’ future needs [1]. NSCs reflect the relationship between C-gain (photosynthesis) and C-loss (respiration and growth) [4, 5]. NSCs are used to endure periods of negative net carbon balance when plants become suddenly defoliated, shaded, and drought-inflicted [6–10]. So, NSCs play a key role in resisting external adverse environmental stress for plants [2, 6]. Carbon (C), nitrogen (N) and phosphorus (P) are the basic elements for plant growth and development. The concentrations of C, N, P in plants reflect nutrient uptake, utilization efficiency and adaptation to the environment stress. Higher N contents are associated with higher leaf area index values, extended photosynthesis duration and greater nutrient uptake [11]. Phosphorus influences photosynthetic assimilation and biomass production in plants [12]. Thus, light intensity affect the leaf photosynthetic capacity, NSC synthesis, and leaf C, N, P content.

Under canopy, light intensity is greatly attenuated before reaching leaf surfaces of seedlings and saplings in the forest understory [13, 14]. Plants have therefore evolved strategies to cope with low light conditions, such as morphological and physiological plasticity, and metabolic adjustments throughout their entire life cycle, especially during the early stages [15, 16]. Shading is especially frequent in the forest understory, and it is therefore likely that allocation to storage would enhance shade tolerance. Some studies predict that more shade-tolerant species should have higher NSCs concentrations [6]. Previous studies showed that soluble sugars and starch content decreased with increasing shading degree [6, 7]. However, Munns (1993) showed that soluble sugars and starch content increased with increasing shading degree [17]. As a result, the NSCs content in different light environment are still controversial, and might be species specific. Shading not only affect the photosynthetic capacity, but also affect the activity of carbon and nitrogen fixation related enzymes, and then affect the content of C and N in plants [18]. Phosphorus involves in several metabolic process and affects biomass production in plants [19]. Thus, the C:N:P variations in leaves are directly affected by shading degree. In recent years, several studies have tested changes in NSCs, the C:N:P stoichiometry in response to different growth conditions, such as temperature, drought, CO_2_ concentrations, nitrogen deposition, and phosphorus addition [20–24]. However, few studies have addressed the effects of shade on species in terms of NSCs and C:N:P stoichiometry.

Thus, we investigated the effects of varying levels of shading on leaf morphology, NSCs and C:N:P stoichiometry in *Cunninghamia lanceolata* (Lamb.) Hook and *Schima superba* Gardn. & Champ – the two most important forest species in subtropical China, which are intended for establishment of mixed species forest. *C. lanceolata*, a fast-growing, high-yielding tree, is one of the most important plantation tree in China [25]. Like other monocultures, the sustainability of *C. lanceolata* plantations is threatened by soil degradation, production loss, biodiversity reduction, and a lack of self-regeneration [26–30]. In order to solve this problem, *S. superba*, a broadleaf tree, is increasingly mixed with *C. lanceolata* stands [26, 27, 31, 32]. Previous studies showed that *C*. *lanceolate* is shade-intolerant tree, in contrast, *S. superba* is shade-tolerant tree [26]. A shift in preference from monoculture plantations to mixed broadleaf-conifer plantations has highlighted the need for research on tree development under management-related variation in light environments. Thus, understanding the morphological and physiological responses to light fluctuations should be useful for determining the proper sequence for introducing species in mixed-species plantations during early post-planting.

The objectives of the study were: 1) to examine responses in leaf traits and NSCs contents to different levels of shading between shade-tolerant and shade-intolerant tree species; 2) to investigate the variations in leaf C, N and P contents, and the C:N:P ratio in response to different levels of shading in shade-tolerant and shade-intolerant tree species; 3) to determine the relationships between leaf NSCs, and C:N:P stoichiometry across shading degrees in shade-tolerant and shade-intolerant tree species. We hypothesized that (1) low light conditions (shade) result in larger leaf area but smaller leaf mass per unit area in shade- tolerant (*S. superba)* than shade-intolerant (*C. lanceolata)* species so as to acquire more light for photosynthesis under low-light environments – the so called carbon gain hypothesis, (2) NSCs concentrations would be higher in shade-tolerant than shade-intolerant species due to low carbon gain in understory while NSC reserves are needed to enhance shade tolerance; and (3) C:N:P stoichiometry varies with shade levels and the response is species-specific due to differences in photosynthetic efficiency and nutrient absorption. To test these hypotheses, we conducted an experiment by altering light intensity along a gradient to determine the differential effects on leaf morphological traits, NSC content and C:N:P stoichiometry in *C. lanceolata* and *S. superba*. We also examined variation in soluble sugar and starch contents as well as leaf C, N, and P contents. Finally, we looked for potential relationships between leaf NSCs, C:N:P stoichiometry, and their combined effects on plant survival mechanisms. The study will provide valuable insights about optimum light conditions for the establishment and growth of both species under mixed planting scheme.

## Results

### Leaf morphological responses to shade

Leaf traits differed significantly (*P* < 0.05) across shade treatments for each species (Table 1). For *S. superba*, leaf length, width, and area were the greatest under 85% shading degree. For *C. lanceolata*, leaf length, width, and area were the greatest under 60% shading degree. Leaf mass per unit area was positively correlated with light for both species.

**Table 1:** Leaf traits of *Cunninghamia lanceolata* and *Schima superba* in response to different shade degrees

Shading degree	*Cunninghamia lanceolata*				*Schima superba*			
	LL (cm)	LW (cm)	LS (cm^2^)	LMA (mg·cm^−2^)	LL (cm)	LW (cm)	LS (cm^2^)	LMA (mg·cm^−2^)
0%*	4.44 ± 0.17d	0.21 ± 0.03b	0.74 ± 0.02d	12.75 ± 0.17a	8.18 ± 0.50c	2.21 ± 0.18d	14.91 ± 0.48d	11.33 ± 0.19a
40%*	4.67 ± 0.15 cd	0.26 ± 0.04ab	0.80 ± 0.03 cd	9.41 ± 0.10b	12.65 ± 0.59b	2.72 ± 0.16c	21.94 ± 0.71c	8.32 ± 0.12b
60%*	6.17 ± 0.14a	0.30 ± 0.03a	1.23 ± 0.03a	9.15 ± 0.14b	13.49 ± 0.57b	3.09 ± 0.17bc	22.62 ± 0.72c	7.69 ± 0.12c
85%*	5.34 ± 0.26b	0.29 ± 0.02ab	0.92 ± 0.04b	9.10 ± 0.25b	18.29 ± 0.78a	3.95 ± 0.16a	43.02 ± 1.82b	6.43 ± 0.16d
95%*	5.16 ± 0.22bc	0.25 ± 0.02ab	0.83 ± 0.02c	5.92 ± 0.10c	17.20 ± 0.67a	3.21 ± 0.12b	32.46 ± 1.51a	5.11 ± 0.08e

Data are represented as means ± SE. Different lowercase letters indicate significant difference (ANOVA, Tukey’s test, *p* < 0.05) among shade treatments within each species. An asterisk after shading degree indicates significant differences between the two species; *LL* Leaf length, *LW* Leaf width, *LS* Leaf size, *LMA* Leaf mass per unit area

### NSCs contents response to shade

**Fig. 1:**
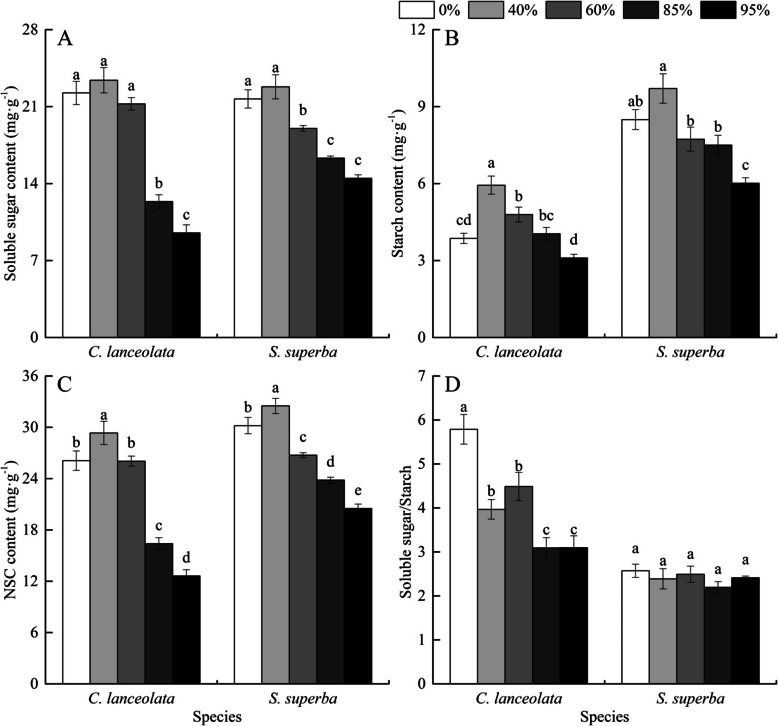
Soluble sugar content (**a**), starch content (**b**), NSC content (**c**), and Soluble sugar/starch ratio (**d**) in leaves of *C. lanceolata* and *S. superba* seedlings under different shading degrees. Bars with different lower letters represent significant differences among shade levels of the same species at 0.05 level

Soluble sugar content, NSC content and soluble sugar/starch ratio varied significantly across all shade levels in both species (Table 2). Soluble sugar content was higher for *C. lanceolata* seedlings exposed to 60, 40 and 0% shading degree than 95 and 85% shading degree, whereas it was higher for *S. superba* seedlings exposed to 40 and 0% shading degree than 95, 85 and 60% shading degree (Fig. 1a). The soluble sugar content was higher for *S. superba* than *C. lanceolata* seedlings exposed to 95 and 85% shading degree. Starch content was higher for *S. superba* than *C. lanceolata* across all shade levels, and the highest starch content was observed in seedlings exposed to 40% shading degree in both species (Fig. 1b). The leaf NSC content was higher in *S. superba* than in *C. lanceolata* under all shade levels, and significantly higher under 0, 85 and 95% shading degree, and in both species NSC content was the highest under 40% shading degree (Fig. 1c). The soluble sugar to starch ratio was larger for *C. lanceolata* than *S. superba* across all levels of shade (Fig. 1d). There was no significant difference in soluble sugar to starch ratio across all levels of shade for *S. superba*. However, *C. lanceolata* seedlings exposed to 0% shading degree had the highest soluble sugar to starch ratio, followed by those exposed to 60 and 40% shading degree and the least being in 95 and 85% shading degree.

**Table 2:** Correlations between leaf NSCs contents and C, N, P content, and C:N:P ratio of *C. lanceolata* and *S. superba* seedlings

Species		Soluble sugar	Starch	NSC (Soluble sugar+Starch)	Soluble sugar/Starch
*C. lanceolata*	C	0.443	−0.136	0.371	0.768^**^
	N	−0.879^**^	−0.788^**^	− 0.903^**^	−0.477^*^
	P	0.248	−0.319	0.168	0.663^*^
	C:N	0.898^**^	0.841^**^	0.929^**^	0.443
	N:P	−0.731^**^	−0.282	−0.692^**^	− 0.762^**^
	C:P	−0.023	0.407	0.045	−0.434
*S. superba*	C	0.555^*^	0.539^*^	0.587^**^	0.024
	N	−0.820^**^	−0.774^**^	− 0.860^**^	−0.082
	P	−0.242	−0.497^*^	− 0.339	0.330
	C:N	0.781^**^	0.735^**^	0.819^**^	0.080
	N:P	−0.725^**^	− 0.436	−0.681^**^	− 0.402
	C:P	0.341	0.583^**^	0.441	−0.306

Date are Pearson correlation coefficients. **Significant at *p* < 0.01, * significant at *p* < 0.05

### Leaf C:N:P stoichiometry responses to shade

**Fig. 2:**
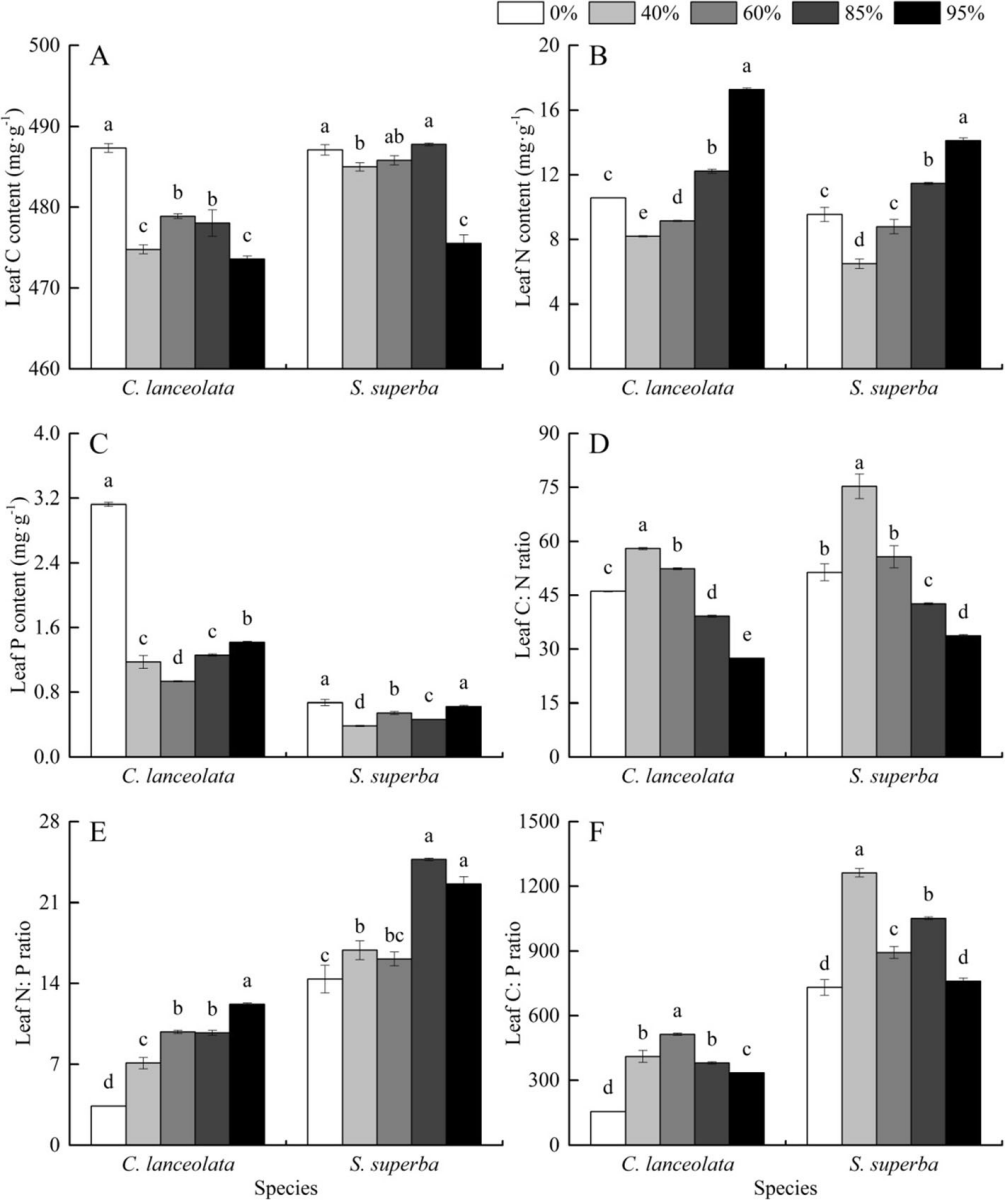
C content (**a**), N content (**b**), P content (**c**), C:N ratio (**d**), N:P ratio (**e**), C:P ratio (**f**) in leaves of *C. lanceolata* and *S. superba* seedlings under different shading degrees. Bars with different lower letters represent significant differences among shade levels of the same species at 0.05 level

Leaf C, N and P contents, as well as C:N:P ratios varied significantly among shade levels in both species. *S. superba* exhibited higher leaf C content than *C. lanceolata* (Fig. 2a). In both species, the leaf N content, contrarily to sugar, NSC, and starch contents that decreased as light reduced, an increasing tendency with increasing shading degree. *C. lanceolata* had higher leaf N content than *S. superba* (Fig. 2b). In both species, the highest leaf N content was observed in 95% shading degree compared to other shading degree. Leaf P content in *C. lanceolata* showed 70% drop from no shading treatment (3.13 ± 0.02 mg·g^− 1^) to 60% shading degree (0.93 ± 0.01 mg·g^− 1^) (Fig. 2c). Leaf P content in *S. superba* was higher under no shading treatment and 95% shade degree than under 40, 60 and 85% shading degree. *C. lanceolata* exhibited higher leaf P content than *S. superba* (Fig. 2c). For both species, leaf C:N ratio decreased with increasing shading degree (Fig. 2d), except no shading treatment. Leaf N:P ratio in both species increased with increasing shading degree (Fig. 2e), leaf N:P ratio of *S. superba* was significantly higher than that of *C. lanceolata*. Leaf C:P ratio of *C. lanceolata* reached the maximum at 60% shading degree, and that of *S. superba* at 40% shading degree (Fig. 2f). *S. superba* had greater leaf C:P ratio than *C. lanceolata* under all shading degree.

### Correlations between NSCs contents and C, N, P contents and C:N:P ratio

Soluble sugar content showed a significantly negative correlation with N content and N:P ratio in both species. Soluble sugar content showed a significantly positive correlation with C:N ratio in *C. lanceolata* and showed significantly positive correlation with C content and C:N ratio in *S. superba* (Table 2). Starch content was significantly negatively correlated with N content in *C. lanceolata*, and with N and P contents in *S. superba*. Starch content was positively correlated with C:N ratio in *C. lanceolata*, and was positively correlated with C content, C:N and C:P ratio in *S. superba.* NSC content positively correlated with C content, C:N, and C:P ratio, while NSC was negatively related to N content and N:P ratio in both species*.* The soluble sugar to starch ratio was positively correlated with C and P content, while it had a negative correlation with N content and N:P ratio in *C. lanceolata.*

## Discussion

The considerable variation in leaf morphology and structure reflects the organ’s phenotypic plasticity [33]. Therefore, leaf characteristics are often used as an indicator of plant acclimation potential and adaptation mechanism [34]. Because excessive irradiance has a detrimental impact on photosynthetic tissues, plants must produce smaller and thicker leaves with higher leaf mass per area under high light conditions. This morphology allows heat dissipation, avoiding damage from overheating and high transpiration rates [35, 36]. Conversely, shaded conditions result in increasing area and decreasing thickness of leaves [26, 37], with low leaf mass per unit area [38]. Increasing leaf area allows plants to acquire more light for photosynthesis [13, 39] and is thus an adaptation to low-light environments [36]. In this study, we observed larger leaf area under 85% shading degree for *S. superba* and under 60% shading degree for *C. lanceolata*. Our findings are in line with previous research on *Elaeagnus angustifolia* leaves, which became smaller and thicker under high light intensity [36].

Furthermore, leaf mass per unit area (LMA) decreased with increasing shading degree in both species. In agreement with our results, *Alocasia macrorrhiza* displays the same adaptations (larger and thinner leaves) to optimize photosynthetic efficiency under low light availability [40]. Shading also resulted in greater LMA for *Citharexylum*, *Dendropanax*, *Fraxinus*, *Quercus*, and *Magnolia* [41]. Interestingly, our study revealed between-species differences in the response of mean leaf area to increasing shading degree. Specifically, mean leaf area was greatest at 60% shading degree in *C. lanceolata*, but at 85% shading degree in *S. superba*. These traits enhanced the ability of *S. superba* to tolerate low light condition (shading) compared with *C. lanceolata*, which concords with a previous study [42]. Our finding is in line with the carbon gain hypothesis that leaf area is higher in shade-tolerant seedlings than in shade-intolerant seedlings [43], and implies that *S. superba* is better adapted to shading.

*C. lanceolata* seedlings have been shown to adapt to shaded conditions through adjusting morphological characteristics [44]. However, seedlings had difficulty maintaining C balance under extremely shaded (95% shading degree) conditions, causing poor growth and survival. The issue of negative C and relatedly NSCs balance under low light is a common problem plants face. For instance, a study made on *Pinus koraiensis* and *Quercus mongolica* demonstrated that low light induced carbohydrate deficiency and therefore high seedling mortality, with none surviving at 1% light intensity [6]. Similarly, under extremely shaded conditions, *Quercus aliena* seedlings had difficulty maintaining C balance and thus experienced mortality [45]. To overcome the lack of an energy source under low light intensity, plants store NSCs to enhance growth and survival [6, 7, 24, 38]. Here, we found that 40% shading degree results in significantly higher soluble sugar, starch, and NSC content for both species. Once under low light intensity, all three variables decreased, presumably as a result of seedlings using their energy stores for growth and also a decrease in C fixation due to light limitation.

Shade-tolerant species should have higher NSCs concentrations than shade-intolerant species [7], because carbon gain was low in understory and NSC reserves are needed to enhance shade tolerance. Other studies also found that shade-tolerant species tend to have greater NSC reserves. For example, the seedlings of palm *Chamaedora elegans* (shade-tolerant species) had higher NSC content than seedling of *Chrysalidocarpus lutescens* (shade-intolerant species) [46, 47]; and the shade-tolerant species *Acer saccharum* seedlings had higher NSC concentrations than seedlings of intermediate light-demanding *Betula alleghaniensis* [48]. In our study, seedlings of shade-tolerant *S. superba* had higher NSC content than shade-intolerant *C. lanceolate*, especially under low light conditions. This result demonstrates that *S. superba* seedlings had an advantage under shaded conditions and, moreover, could flexibly adjust to a vast range of shade levels. In terms of mechanism, exposure to high light intensity would result in greater C gain than demand, leading to NSCs storage [38, 49]. Once light becomes a limiting resource, plants will mobilize NSCs to support growth and survival [50]. Under 85% shading, growth in height, diameter and biomass production of *S. superba* were considerable higher than other shading treatments (Data not shown). The results support our hypothesis that *S. superba* produces more NSCs under low light condition than *C. lanceolata*. This finding agrees with a previous study that demonstrated that shade-tolerant species exhibit higher NSCs content than shade-intolerant species [7].

Both genetic and environmental factors influence plant nutrient uptake, as demonstrated by interspecific differences, along with intraspecific differences under various habitats [51]. In our study, *S. superba* and *C. lanceolata* produce C during photosynthesis and absorb N and P differently under varying shading degree, suggesting species-specific strategies in balancing nutritional metabolism and adapting to environmental stress. Both species had higher C content under intermediate shade condition (40–60% to full light availability), likely due to strong photosynthetic efficiency resulting in heightened synthesis of organic matter and C accumulation. Importantly C content was significantly larger in *S. superba* than in *C. lanceolata*. Given previous research linking higher C content with greater photosynthetic efficiency and resilience to adverse environments [52], our findings imply that *S. superba* is better adapted to low light condition than *C. lanceolata*. Our results are consistent with previous studies demonstrating that shade-tolerant plants have higher NSCs accumulation and C pool than shade-intolerant plants [7, 24], this is because their photosynthetic machinery is adapted to be more efficient in the low light condition and store more C than plants that are not adapted their photosystems to low light. Higher P and N contents in both species were observed under no shading treatment and 95% shading degree, respectively. These results support that the adaptive strategy to shade might be species specific. P and N are essential macro-elements for plant growth and development, which participate in a number of metabolic processes, such as photosynthetic phosphorylation, ATP production, the production and export of triose-P and ribulose-1, 5-bisphosphate regeneration as well as synthesis of amino acids [53]. This outcome is the vigorous growth under strong photosynthetic ability in full sunlight, leading to greater requirements for proteins and nucleic acids. On the contrary, seedlings of both species may use more N resources to synthesize light-trapping proteins under low light intensity. This is further evidenced in our study that NSC content was negatively correlated with N content and N:P ratio in both species, whereas a positive correlation was observed between NSC and C:N ratio in *C. lanceolata* and with C content and C:N ratio in *S. superba.* Our findings are corroborated by previous research showing that plants growing under low light condition will have increased leaf N content and allocate more N to photosynthetic pigments. We observed higher chlorophyll a content in *S. superba* than in *C. lanceolata* (data not shown). Due to the prevention of photo-damage, this strategy increases light use efficiency and maintain normal photosynthetic function [54]. The findings give credence to our results that C:N:P stoichiometry varies with shade levels might be species-specific. As a whole, the findings have greater implication for establishment and maintenance of mixed species stand. *S. superba* is better adapted to low light intensity (shade tolerant), thus it would be advisable to plant *S. superba* later once the canopy of *C. lanceolata* is well developed but allowing enough sunlight (up to 40%). Conversely, thinning of dense stands of *C. lanceolata* to allow sufficient light to reach the understory would be recommended to expedite the natural regeneration and subsequent growth of *S. superba*.

## Conclusions

The results demonstrate that shading significantly affected foliage morphology, leaf NSC content, and C, N, P stoichiometry in shade-intolerant species *C. lanceolata* and shade-tolerant species *S. superba* seedlings. In general, both species showed a decrease in NSC, soluble sugar and starch content with increasing shading degree. However, *S. superba* had higher NSC content than *C. lanceolate*, especially under low light conditions. These results imply a decrease in photosynthesis efficiency in *C. lanceolata* with increasing shading and suggest that carbohydrate storage is especially important for species that regenerate in persistently shady habitats. Highly significant correlations were found between leaf NSC variables and C, N, P content and C:N:P ratio in *C. lanceolate* and *S. superba*. It was likely that the dynamic trade-off of photosynthesis products exists between leaf NSCs and C:N:P stoichiometry. Our results improve our understanding of the balance of leaf C, N, P components and NSCs contents in shade-tolerant and shade-intolerant plants. In addition, the findings have greater implication for establishment of mixed species stand. As *S. superba* is better adapted to low light condition (shade tolerant), it would be advisable to plant *S. superba* later once the canopy of *C. lanceolata* is well developed but allowing enough sunlight (up to 40% light transmittance). Conversely, in dense stands of *C. lanceolata*, thinning to allow sufficient light to reach the understory would be recommended to expedite the natural regeneration and subsequent growth of *S. superba* as we observed better growth of *S. superba* under low light condition.

## Materials and methods

### Experimental design and treatments

The pot experiment was conducted in a flat, open area at the Fujian Agriculture and Forestry University. Five shade levels were created, i.e., 0% (control, no-shading), 40% shaded (60% irradiance), 60% shaded (40% irradiance), 85% shaded (15% irradiance), 95% shaded (5% irradiance). Each shading degree was created using frame covered with black nylon shade cloth of differing mesh size (0% shaded did not use shade cloth) [55]. The frames were 2.0 m high, 6.0 m × 2.5 m in length and width, and were placed parallel to the sun’s daily track to minimize spatiotemporal variation in solar radiation. The light intensity in each shading degree treatment was measured with two light meters (Hipoint HP350, Taiwan, China, and Red/Far-red Sensor, Skye Instruments Ltd., UK) during clear day (see Appendix S1).

In July 2016, *C. lanceolata* and *S. superba* seedlings were purchased from a container nursery in Zhangping Wuyi Forest Farm, Fujian, China. Purchased seedlings were transplanted to pots containing a mixture of peat soil and vermiculite (2:1 w/w) and were grown for 1 month in a greenhouse at the experimental site. Fertilizer was not added during the experiment period. In August 2016, well-developed seedlings of uniform height (*C. lanceolata*: 18.49 ± 1.97 cm, *S. superba*: 27.40 ± 1.19 cm) were selected and randomly divided into five groups. Each group comprised four seedlings per species and was assigned to each of shading degree treatment. Individual seedling pots were treated as replicates and randomly positioned to ensure each obtained similar light irradiation with no mutual shading. Pots were rotated weekly to ensure homogeneous conditions. Weeds were periodically cleared from the experimental plot and seedlings were watered 2–3 times weekly.

### Leaf morphology measurements

All plants were maintained under their assigned shade levels for 1 y. To estimate mean leaf area, 10 healthy and fully expanded green leaves were randomly collected from seedlings of comparable height in the same pot. In order to keep the leaves fresh, leaf samples were placed in ice and immediately taken to the laboratory for further analysis. Individual leaf area (cm^2^) was determined with a portable leaf area meter (Yaxin-1241, Shanghai, China). Leaves were then individually placed in paper bags and oven-dried for 30 min at 105 °C, followed by at least 24 h at 80 °C. Upon reaching a constant dry mass, the dry mass of each leaf was determined. Leaf mass per unit area (LMA, mg·cm^− 2^) was computed as the oven-dry mass per leaf divided by the corresponding area.

### Measurements of NSCs

At the end of the experiment in August 2017, leaves were randomly collected from seedlings of both species across all shade treatments, cleaned with distilled water, and grounded to powder. 0.2 g of the ground sample were mixed with 5 mL of distilled water in a test tube. After 30 min in a boiling water bath, the supernatant was collected. This process was repeated twice to ensure complete sugar extraction. The two extracts were collected in a centrifuge tube and distilled water was then added to achieve a 25 mL constant volume. Thereafter, sediments from the soluble sugar extraction were dried and then the perchloric acid was added to extract starch. Soluble sugar and starch contents were determined using the anthrone colorimetric method. Absorbance at 630 nm was measured to calculate soluble sugar and starch contents according to the glucose standard curve. Non-structural carbohydrates content was calculated as the sum of soluble sugar and starch content. The analysis was replicated four times per treatment.

### Measurements of leaf C, N, P

At the end of the experiment, all leaves of the same replicate seedling under the same treatment were collected, and then grounded into uniformly fine powder, and sieved with a 1 mm mesh before chemical analysis. Total C and N content (mg·g^− 1^, dry mass basis) were measured via dry combustion using an elemental analyzer (VARIO MAX CN; Elementary, Germany). Total P concentration (mg·L^− 1^) was determined with ICP-OES (Optima 8000, PerkinElmer) after H_2_SO_4_-HClO_4_ solution digestion and dilution. After converting to mg·g^− 1^, the C:N, C:P and N:P ratios were calculated as content ratio. All chemical analyses were replicated four times per light treatment and species.

### Statistical analysis

One-way ANOVA was performed for each species separately to test the significant effect of shading on leaf morphology, NSCs contents, and C, N, P content, and C:N:P stoichiometry. Pearson’s correlation analysis was performed to examine the relationship between NSCs contents, and C, N, P content and C:N:P ratio. Data are presented as means ± SE for different shade treatments and species. Statistical significance was set at *p* < 0.05. All statistical analyses were performed in SPSS version 20.0 for Windows (SPSS Inc., Chicago, IL, USA).

## Supplementary information

**Additional file 1: Appendix S1.** Light conditions in different shade treatments (mean ± SE). Different letters indicate significant differences in light conditions across shade treatments.

**Additional file 2.**

## Data Availability

Data are made available as supplementary material.

## Abbreviations

NSC: Non-structural carbohydrate
C: Carbon
N: Nitrogen
P: Phosphorus
LMA: Leaf mass per unit area
LL: Leaf length
LW: Leaf width
LS: Leaf size
