# Supplementary material for: Responses of leaf morphology, NSCs contents and C:N:P stoichiometry of Cunninghamia lanceolata and Schima superba to shading
Source: BMC Plant Biol. 2020 Jul 29;20:354. doi: 10.1186/s12870-020-02556-4 (PMC7391624; doi:10.1186/s12870-020-02556-4)
Supplement: Supplementary file 1 — Additional file 1: Appendix S1. Light conditions in different shade treatments (mean ± SE). Different letters indicate significant differences in light conditions across shade treatments. [file 12870_2020_2556_MOESM1_ESM.docx]

Appendix S1. Light conditions in different shade treatments (mean ± SE). Different letters indicate significant differences in light conditions across shade treatments.

| Shading degree | Illuminance/(Lux) | Photosynthetic Photon Flux Density/(μmol·m^-2^·s^-1^) | Red/Far red ratio |
| --- | --- | --- | --- |
| 0% | 61860.11±1170.73a | 1101.88±22.81a | 1.07±0.01a |
| 40% | 37214.13±885.93b | 669.76±32.12b | 1.07±0.01a |
| 60% | 24805.29±424.82c | 453.88±16.17c | 1.06±0.01a |
| 85% | 9357.80±374.01d | 166.91±6.62d | 1.06±0.01a |
| 95% | 2889.60±89.48e | 51.60±1.59e | 1.06±0.02a |
